# Prevalence and Associated Factors of Intestinal Parasites among Food Handlers Working in Food Service Establishments in Northwest Ethiopia, 2022

**DOI:** 10.1155/2023/3230139

**Published:** 2023-05-18

**Authors:** Hailegebriel Wondimu, Mestawut Mihret

**Affiliations:** ^1^Department of Medical Laboratory, Debre Tabor Health Science College, Debre Tabor, Ethiopia; ^2^Department of Nursing, Debre Tabor Health Science College, Debre Tabor, Ethiopia

## Abstract

**Background:**

As in most of African countries, intestinal parasites have been widely distributed in Ethiopia and are among the 10 top causes of morbidity and mortality nationwide. Statistics for food-borne illness in various industrialized countries show that up to 60% of cases may be caused by poor food handling techniques and by contaminated food served in food service establishments. Epidemiological information on the prevalence of various intestinal parasitic infections in different regions/localities is a prerequisite to develop appropriate strategies.

**Objective:**

This study aimed to determine the magnitude of intestinal parasites among food handlers working in different food service establishments in Gondar city.

**Methods:**

A cross-sectional study was conducted with food handlers working in different food service establishments in Gondar city. Stool samples were collected from 350 food handlers and processed using the formol-ether concentration method and then microscopically examined for intestinal parasitic infections. Pre-tested and structured questionnaire was used to study the socio-demographic characteristics of food handlers. Chi-square test and *p*-value were used to assess the associations between risk factors and the parasite isolation rate. The *p*-value ≤0.05 was considered as statistically significant.

**Results:**

Of the 350 food handlers, 160 (45.71%) had parasites. Among the isolated parasites, *Ascaris lumbricoides* was found to be the most prevalent parasite 35.63%, followed by hookworm 19.38%, *Entamoeba histolytica/dispar* 16.25%, *Trichuris trichiura* 10.00%, *Strongyloides stercoralis* 8.13%, *Schistosoma mansoni* 6.88%, and *Cystoisospora belli, Hymenolepis nana*, and *Taenia* species each accounting 1.25%.

**Conclusion:**

The result of the study indicated that the magnitude of intestinal parasitosis among food handlers working at different levels of food establishments in Gondar, Ethiopia, was found to be high. Being at lower educational level and inactive role of the town's municipality are determined as a risk factor for parasitic positivity of food handlers.

## 1. Introduction

Food-borne diseases are caused by bacteria, viruses, fungi, and parasites. Intestinal parasitosis refers to a group of diseases caused by one or more species of protozoa and helminths. These parasites are responsible for the major share of morbidity and mortality in a community where there is overcrowding, poor environmental sanitation, and poor personal hygienic practices, economic and social conditions also affect the distribution of human parasites [1–4].

Globally, due to intestinal parasitic infections, around 3.5 billion people are affected and more than 200,000 deaths are reported annually. Around 50,000 deaths yearly are caused by intestinal parasites in Ethiopia [5, 6].

As in most of African countries, intestinal parasites have been widely distributed in Ethiopia and are among the 10 top causes of morbidity and mortality nationwide [3]. According to the Ministry of Health of Ethiopia, intestinal parasitism accounts for 8.5% of all male and 10.4% of all female outpatient infections. Prevalence rates higher than 70% and high rate of multiple infections in those infected individuals have been reported from many parts of the country [7]. Although the prevalence rates of individual parasites vary considerably latitudinal in different parts of the country, several studies show that *Ascaris lumbricoides* is the most prevalent intestinal parasite, followed by *Trichuris trichiura*, a hookworm, and *Strongyloides stercoralis* [8]. The prevalence of *A. lumbricoides* infection was 29% in the highlands, 35% in the temperate areas, and 38% in the lowlands. The prevalence of hookworm infection was highest in the lowlands 24%, followed by highlands 17% and temperate 15% areas, and the differences were significant. *T. trichiura* infection exhibited similar prevalence in all altitudinal regions (13% on average). Earlier work showed that intestinal parasitism had prevalence rates of 20–70% in Gondar region [9]. High prevalence of intestinal parasitic infections affects the health status of individuals, mainly affecting physical and mental development, causing malnutrition, anemia, stunting, cognitive impairment, lowered educational achievement, and interfering with productivity [4].

Parasitic infections, especially helminthic infections, are clearly persistent within human communities in endemic areas and hence remain infectious for other healthy individuals [10].

Transmission of intestinal parasites is affected directly or indirectly through faecally contaminated objects such as food, water, soil, and fingers [2].

Accordingly, food handlers with poor personal hygiene working in food service establishments could be potential sources of infection for many of the intestinal helminths and protozoa [11]. Food handlers who harbor and excrete intestinal parasites may contaminate food from their faeces to their hands and then to the food process, and healthy person may be infected by eating contaminated food [4].

Statistics for food-borne illness in various industrialized countries show that up to 60% of cases may be caused by poor food handling techniques and by contaminated food served in food service establishments [12].

Hence, in order to implement safe food processing up to consumption practices in food service establishments, determining the magnitude of intestinal parasitosis among food handlers working in different levels of food service establishments is mandatory for the most practical and economical control and prevention measures to be undertaken.

## 2. Methods and Materials

### 2.1. Study Area and Period

The study was conducted in Amhara Regional State, Central Gondar Zone, Gondar, from June to September 2022. The city of Gondar is situated in Northwestern part of Ethiopia, Amhara Regional State. It is at 12°3′N latitude and 37°28′E. Gondar is located 727 km from Addis Ababa, the capital city of Ethiopia, and 120 km from Bahir Dar, the capital city of Amhara National Regional State. Gondar has a total area of 192.3 km^2^ with undulating mountainous topography. According to the Central Statistics Agency (CSA) population projection (2013), the population of Gondar was estimated to be 360,600 in 2017 [13].

### 2.2. Study Design

A descriptive institution-based cross-sectional study design was conducted.

### 2.3. Source Population

All individuals employed as food handlers to work in food service establishments in Gondar.

### 2.4. Study Population

All randomly selected food handlers working in food service establishments in Gondar city during the study period.

### 2.5. Eligibility Criteria

Food handlers working in food service establishments in Gondar city were included in the study. However, those food handlers who were ill, took any antiparasitic drugs at the time of the study or 2 weeks prior to the study, and unable to give a response were excluded.

### 2.6. Sample Size Determination

The sample size was based on the assumption of the proportion of pooled prevalence estimate of intestinal parasites among food handlers of food service establishments in Ethiopia, which was 33.6% [14].

With 5% marginal error and 95% confidence interval of certainty (alpha (*a*) = 0.05), the actual sample size for the study is computed using one sample population proportion formula as indicated below:

(1) $$\begin{array}{c} n=\frac{{\left(Z^{a}/2\right)}^{2}pq}{d^{2}}, \end{array}$$

where: *n* = sample size, *p* = proportion of women who knew about obstetric danger signs, *q* = proportion of failure (1 − *p*), *Z^a^*/2 = critical value 1.96, *d* = precision (marginal error) = 0.05, *N* = total sample size.

Thus, the sample size was

(2) $$\begin{array}{c} n=\frac{{\left(1.96\right)}^{2}\times 0.34\times 0.66}{{\left(0.05\right)}^{2}}=345, \end{array}$$

(3) $$\begin{array}{c} N=n+n\times 10\%\left(\text{contingency}\right)=345+35=380. \end{array}$$

Therefore, 380 food handlers working in food service establishments in Gondar town were enrolled in the study.

### 2.7. Sampling Procedure

The lists of food service establishments were obtained from Industry Investment and development office of Gondar town. Each level of food service establishment was considered as a cluster, and the number of clusters to be studied was determined using a probability proportional to the size technique for each stratum. Simple random sampling technique was used to select the number of subjects from each cluster to be studied. There were about 1043 food handlers working in different levels of food service establishments. Out of which 380 were the sample size for this study, 29 from bars and restaurants, 214 from hotels, 93 from restaurants, and 44 from tea and breakfast rooms. The questionnaire was pretested by 10% of samples from food handlers in a nearby town of the study area, Maksegnit. Pre-tested and structured questionnaires were used to produce general information on socio-demographic characteristics of food handlers. They were asked for their views on the determinants of hygienic and sanitary conditions within the establishments they work. Stool samples were taken from the selected food handlers using leak-proof plastic caps containing 8 ml of 10% formalin.

### 2.8. Stool Sample Collection and Examination

From each study subject, a sufficient amount of fresh stool samples were collected using small clean plastic cups containing 8 ml of 10% formalin preservative. All specimens were processed by the formol-ether concentration technique, which is considered as the most sensitive for most intestinal helminths and protozoan cysts [15]. About 1 g of stool was added to a clean 15 ml conical test tube containing 7 ml of 10% formal saline. The stool was gently suspended with formal saline using an applicator stick. The suspension was filtered through a sieve into a second centrifuge tube. After adding 3 ml of diethyl ether, contents in the second tube were centrifuged at medium speed (2500 rpm) for 5 minutes. The supernatant was poured off, and a smear on a clean slide was prepared from the sediment and covered with a clean cover slip. The preparation was examined in the same way as that of the direct saline method. Negative results were reported after assessing the whole smear under the 10× objective [15]. Investigators supervised all aspects of data collection and laboratory procedures.

### 2.9. Data Analysis

Data were analyzed using SPSS version 21. Chi-square test and *p*-value were used to assess the association between risk factors and the parasite isolation rate. The *p*-value ≤0.05 was considered as statistically significant.

### 2.10. Ethical Consideration

Ethical approval of the research was obtained from Ethical Review Committee of School of Biomedical and Laboratory Sciences, College of Medicine and Health Sciences, University of Gondar, and an official letter was directed to Gondar town municipality. Written informed consent was obtained from the study participants. Food handlers with parasitic infections were treated in accordance with the Ethiopian national treatment guideline. Information obtained in any course of the study was kept confidential.

## 3. Results

### 3.1. Socio-Demographic Characteristics

Out of the total selected 380 food handlers in all establishments, 350 of them responded making an overall coverage of 92.11%. Majority of the respondents were less than 30 years, 307 (87.86%) with a mean age of 30, female 319 (91.14%), with an informal educational level 162 (46.29%), and the average income of food handlers were 2000 Ethiopian Birr per month (Table 1).

### 3.2. Prevalence of Intestinal Parasitosis

From the total 350 sampled stool specimens, 160 (45.71%) were positive for the parasite. Among the isolated parasites, *A. lumbricoides* was found to be the most prevalent parasite 57 (35.63%), followed by hookworm 31 (19.38%), *Entamoeba histolytica/dispar* 26 (16.25%), *T. trichiura* 16 (10.00%), *S. stercoralis* 13 (8.13%), *Schistosoma mansoni* 11 (6.88%), and *Cystoisospora belli*, *Hymenolepis nana*, and *Taenia* species each accounting 2 (1.25%) (Table 2).

Of the four types of food service establishments, hotels showed a greater prevalence of intestinal parasites among food handlers 60 (17.14%), followed by restaurants 51 (14.57%), tea and breakfast rooms 42 (12.00%), and bars and restaurants 7 (2.00%) (Table 2).

### 3.3. Associated Factors with Intestinal Parasitosis among Food Handlers

In the data analysis, based on the Chi-square test and *p*-value to assess the association of risk factors with the intestinal parasitic infection rate, only the educational levels of study participants and municipality role were considered risk factors. The *p*-value ≤0.05 was used as a cut-off point for the evaluation of association (Table 3).

## 4. Discussion

Epidemiological study on the prevalence of infection of intestinal parasites in different localities is a primary objective to identify high risk communities and formulate an appropriate intervention [16]. In line with this view, the current study attempted to assess the prevalence of intestinal parasitic infections in food handlers working in different types of food service establishments in Gondar town. The results of this study showed the occurrence of several intestinal parasites of public health importance among food handlers working in different types of food service establishments in Gondar town.

Absent and/or low prevalence of intestinal parasites might be due to the single technique used, which is considered as the limitation of the study. Specific methods such as adhesive scotch tape for *Enterobius vermicularis* [17] and Kato–Katz technique for most intestinal parasites were also good [18]. As a result, a much greater rate of parasites would have been found if these methods in combination were used in this study. A review of the literatures reveals very few investigations of intestinal parasites in those food handlers working in different food service establishments in different towns.

Earlier studies showed that intestinal parasites had a prevalence rate of 20–70% in Gondar region [9, 19], and the result of the current study was also found within this range of 45.71%. As well, intestinal parasites detected in the stools of the food handlers were *A. lumbricoides*, hookworm, *E. histolytica/dispar*, *T. trichiura*, *S. stercoralis*, *S. mansoni*, *C. belli, H. nana,* and *Taenia* species, which is supported by the result of the study conducted by Andargie et al. [11]. This might be due to similarity in the study area.

The magnitude of intestinal parasites obtained in this study was higher than the results of studies conducted among food handlers in Jimma 33% [20] and Wolaita Sodo 23.6% [21]. This might be due to a variation in socioeconomic and cultural conditions as the above studies were conducted in different regions of the country. Is also supported by the report of this study as the higher prevalence of intestinal parasites has an association with the educational level of study participants and municipality role. For instance, the associated risk factors in this study showed the highest parasitic infections in illiterate food handlers and absence of the municipality role with statistical difference (*p* < 0.05). Therefore, being an illiterate and inactive role of the municipality were determined as associated factors for parasitic positivity of food handlers. The planning to reduce parasitic infections among food handlers should focus to provide the knowledge of parasitic infections and how to protect themselves and to arrange the role of municipality is needed.

## 5. Conclusion

The result of the study indicated that the magnitude of intestinal parasitosis among food handlers working at different levels of food establishments in Gondar town, Ethiopia, was found to be high (45.71%). Being lower in educational level and absence of the municipality role in food service establishments are risk factors. Therefore, it is important to promote and educate food handlers, and there is a need to have an active role of the town municipality such as provision of training on personal and environmental hygiene, waste management, food hygiene and safety, and establish a system like regular inspection of the establishments, sanitation, and medical check-up of food handlers.

## Figures and Tables

**Table 1:** Socio-demographic characteristics of food handlers working in different food service establishments in Gondar.

Variables	Level	Hotels *n* (%)	Restaurant *n* (%)	Bar and restaurant *n* (%)	Tea and breakfast *n* (%)	Total *n* (%)
Age	≤20	2 (10.53)	5 (26.32)	4 (21.05)	8 (42.11)	19 (100.00)
	21–30	71 (47.33)	59 (39.33)	7 (4.67)	13 (8.67)	150 (100.00)
	31–40	54 (35.53)	60 (39.47)	9 (5.92)	29 (19.08)	152 (100.00)
	41–50	6 (31.58)	2 (10.53)	0 (0.00)	11 (57.89)	19 (100.00)
	>50	7 (70.00)	0 (0.00)	0 (0.00)	3 (30.00)	10 (100.00)
Sex	Female	127 (39.81)	120 (37.62)	18 (5.64)	54 (16.93)	319 (100.00)
	Male	13 (41.94)	6 (19.35)	2 (6.45)	10 (32.26)	31 (100.00)
Educational level	Illiterate	55 (33.95)	72 (44.44)	15 (9.260)	20 (12.350)	162 (100.00)
	Primary level	53 (42.40)	39 (31.20)	5 (4.00)	28 (22.40)	125 (100.00)
	Secondary and above	32 (50.79)	15 (23.81)	0 (0.000)	16 (25.40)	63 (100.00)
Marital status	Married	46 (38.33)	48 (40.00)	13 (10.83)	13 (10.83)	120 (100.00)
	Single	114 (45.60)	78 (31.20)	7 (2.80)	51 (20.400)	250 (100.00)
Income per month in Birr	<1000	0 (0.00)	1 (33.33)	0 (0)	2 (66.67)	3 (100.00)
	1001–2000	83 (41.92)	63 (31.82)	11 (5.56)	41 (20.71)	198 (100.00)
	2001–4000	46 (35.380)	58 (44.62)	7 (5.38)	19 (14.62)	130 (100.00)
	>4000	11 (57.890)	4 (21.05)	2 (10.53)	2 (10.53)	19 (100.00)
Type of latrine	Private	140 (41.30)	124 (36.58)	20 (5.90)	55 (16.22)	339 (100.00)
	Public	0 (0.00)	2 (18.18)	0 (0.00)	9 (81.82)	11 (100.00)
Water source	Pipe	140 (40.00)	126 (36.00)	20 (5.71)	64 (18.29)	350 (100.00)
Food service utensils washing style	By water with detergents	140 (40.00)	126 (36.00)	20 (5.71)	64 (18.29)	350 (100.00)
Municipality role in waste disposal	Present	70 (43.21)	45 (27.78)	20 (12.35)	27 (16.67)	162 (100.00)
	Absent	70 (37.23)	81 (43.09)	0 (0.00)	37 (19.68)	188 (100.00)

**Table 2:** Parasitological stool examination results of food handlers working in different food service establishments in Gondar.

Types of intestinal parasites	Hotels *n* (%)	Restaurant *n* (%)	Tea and breakfast *n* (%)	Bar and restaurant *n* (%)	Total *n* (%)
Number of infections from the total sample *n* (%)	60 (17.14)	51 (14.57)	42 (12.00)	7 (2.00)	160 (45.71)
Protozoa					
*E. histolytica*	6 (10.00)	7 (13.73)	10 (23.81)	3 (42.86)	26 (16.25)
*C. belli*	2 (3.33)	0 (0.00)	0 (0.00)	0 (0.00)	2 (1.25)
Helminths					
*A. lumbricoides*	29 (48.33)	18 (35.29)	8 (19.05)	2 (28.57)	57 (35.63)
Hookworm	7 (11.67)	11 (21.57)	11 (26.19)	2 (28.57)	31 (19.38)
*T. trichiura*	10 (16.67)	1 (1.96)	5 (11.90)	0 (0.00)	16 (10.00)
*S. stercoralis*	3 (5.00)	3 (5.88)	7 (16.67)	0 (0.00)	13 (8.13)
*S. mansoni*	3 (5.00)	7 (13.73)	1 (2.38)	0 (0.00)	11 (6.88)
*H. nana*	0 (0.00)	2 (3.92)	0 (0.00)	0 (0.00)	2 (1.25)
*Taenia* species	0 (0.00)	2 (3.92)	0 (0.00)	0 (0.00)	2 (1.25)
Total	60 (100)	51 (100.00)	42 (100.00)	7 (100.00)	160 (100.00)

**Table 3:** Factors having an association with parasitic positivity of food handlers working in food service establishments in Gondar.

Variables	Parasitic infections		𝜒^2^ (*p*-value)
	Positive *n* (%)	Negative *n* (%)	
Educational level			
Without formal educations	87 (54.37)	75 (39.47)	3.71 (<0.001)
Primary level	58 (36.25)	67 (35.26)	
Secondary and above	15 (9.38)	48 (25.27)	
Municipality role			
Absent	107 (66.87)	81 (42.63)	2.72 (<0.001)
Present	53 (33.13)	109 (57.37)	
Total	160	190	

## Data Availability

The data used to support the findings of this study are available from the corresponding author upon request.
